# Development and evaluation of the focused assessment of sonographic pathologies in the intensive care unit (FASP-ICU) protocol

**DOI:** 10.1186/s13054-021-03811-2

**Published:** 2021-11-24

**Authors:** Stefan Schmidt, Jana-Katharina Dieks, Michael Quintel, Onnen Moerer

**Affiliations:** 1 Department of Anesthesiology, Emergency and Intensive Care Medicine, University Hospital Goettingen, Georg August University, Robert-Koch-Str. 40, 37075 Goettingen, Germany; 2 Department of Pediatric Cardiology and Pediatric Intensive Care Medicine, University Hospital Goettingen, Georg August University, Robert-Koch-Str. 40, 37075 Goettingen, Germany

**Keywords:** Sonography, Ultrasound, General critical care ultrasound, Whole-body ultrasound, Critical care echocardiography, Intensive care medicine, Critical care

## Abstract

**Background:**

The use of ultrasonography in the intensive care unit (ICU) is steadily increasing but is usually restricted to examinations of single organs or organ systems. In this study, we combine the ultrasound approaches the most relevant to ICU to design a whole-body ultrasound (WBU) protocol. Recommendations and training schemes for WBU are sparse and lack conclusive evidence. Our aim was therefore to define the range and prevalence of abnormalities detectable by WBU to develop a simple and fast bedside examination protocol, and to evaluate the value of routine surveillance WBU in ICU patients.

**Methods:**

A protocol for focused assessments of sonographic abnormalities of the ocular, vascular, pulmonary, cardiac and abdominal systems was developed to evaluate 99 predefined sonographic entities on the day of admission and on days 3, 6, 10 and 15 of the ICU admission. The study was a clinical prospective single-center trial in 111 consecutive patients admitted to the surgical ICUs of a tertiary university hospital.

**Results:**

A total of 3003 abnormalities demonstrable by sonography were detected in 1275 individual scans of organ systems and 4395 individual single-organ examinations. The rate of previously undetected abnormalities ranged from 6.4 ± 4.2 on the day of admission to 2.9 ± 1.8 on day 15. Based on the sonographic findings, intensive care therapy was altered following 45.1% of examinations. Mean examination time was 18.7 ± 3.2 min, or 1.6 invested minutes per detected abnormality.

**Conclusions:**

Performing the WBU protocol led to therapy changes in 45.1% of the time. Detected sonographic abnormalities showed a high rate of change in the course of the serial assessments, underlining the value of routine ultrasound examinations in the ICU.

*Trial registration* The study was registered in the German Clinical Trials Register (DRKS, 7 April 2017; retrospectively registered) under the identifier DRKS00010428.

**Supplementary Information:**

The online version contains supplementary material available at 10.1186/s13054-021-03811-2.

## Background

Bedside sonographic evaluations performed by intensivists are increasingly used in daily routine practice in the intensive care unit (ICU) [1]. Based on point-of-care ultrasound (POCUS) protocols that have been adapted from non-ICU settings, specific techniques have been developed for use in critically ill patients in the ICU and emergency department (ED) settings. Even some ultrasound techniques that were previously considered advanced are now regarded as mandatory critical care core ultrasound competencies or are at least recognized as being acceptable and possible within the ICU [2]. Apart from being relatively noninvasive and of low cost, ultrasound assessments are regarded as a very promising way to further improve the outcome of ICU and ED patients. Ultrasonography has been identified as one prerequisite for practicing individualized patient care and, when combined with other measures, for precision medicine within the ICU [3].

While recommendations for ultrasound use in the ICU are currently based on a growing body of scientific evidence, there is only very limited evidence available regarding whole-body ultrasound (WBU) and, more importantly, a lack of agreement on what competencies are required [4]. The first aim of our study was therefore to define the range and prevalence of sonographically detectable abnormalities, by performing serial WBU examinations in ICU patients. In previous studies, predominantly only single-organ systems were evaluated, but there are currently no easy-to-follow WBU protocols. The second aim of our study was to develop and evaluate the first simple and fast bedside WBU protocol, with 99 predefined pathologic entities, that can be employed to evaluate ICU patients by all intensivists trained in POCUS.

The diagnostic value of *acute* ultrasound or echocardiographic examinations in the ED and the ICU has been shown previously [5–8]. Furthermore, the impact of surveillance critical care echocardiography in the ICU has recently been demonstrated [9]. In this study, a critical care echocardiographic examination was performed on day 3 after admission to the ICU to detect cardiac abnormalities at an early stage rather than having to perform echocardiography once the individual hemodynamics had acutely deteriorated. However, little is known about the value of surveillance or serial ultrasound examinations of multiple-organ systems in the ICU. One available study focused on examinations on the day of ICU admission [10]. To address this lack of data, the third aim of our study was to evaluate a routine WBU protocol performed as a surveillance measure on five occasions over the first 15 days of the ICU stay. Finally, we evaluated the benefit of routine and potentially preventive WBU evaluations in the ICU and recorded changes in the intensive care therapy implemented as a direct consequence of the sonographic findings.

## Methods

### Patient selection, enrollment and institutional approval

One hundred and eleven consecutive patients were studied in two anesthesiology-supervised surgical ICUs (42 beds) at the tertiary university hospital of Goettingen in Germany (1460 beds). Patients younger than 18 years of age and palliative patients were excluded from the study. The study was approved by the local ethics committee (ethics proposal Universitatsmedizin Goettingen 25/6/13, 12 August 2014). Written informed consent was obtained from the participating patients or their legal guardians. The study was registered in the German Clinical Trials Register (DRKS, 7 April 2017) under the identifier DRKS00010428.

### Image acquisition and study protocol

Patients underwent imaging according to the focused assessment of sonographic abnormalities in the intensive care unit (FASP-ICU) protocol. They received ocular, vascular, pulmonary, cardiac and abdominal ultrasound examinations on admission to ICU and on days 3, 6, 10 and 15 of their ICU stay. Sonographically detectable abnormalities were predefined (FASP-ICU protocol, Additional file 1), and 99 different entities were assessed. Predefined abnormalities were selected on the basis of previous ultrasound examinations and experience in critical care, on expected prevalence, and on expected reliable reproducibility of the required ultrasound technique by intensivists with skills already established or with skills that could easily be added into routine practice. Detected abnormalities that were not predefined were recorded as additional findings. The duration of the examination from start until the end of image acquisition was recorded. A single expert examiner, trained in internal medicine and cardiology, as well as in anesthesiology and intensive care medicine, performed all ultrasound examinations. A second reviewer had full access to all obtained images and reviewed the diagnoses for verification. This reviewer was initially blinded to the diagnoses made by the first examiner; agreement between the reviewer and the first examiner was reached by following a previously defined protocol. Attending intensivists also had access to all obtained images at all times.

A General Electric (GE) Healthcare Vivid S5 machine, equipped with a phased array adult 1.5–3.6 MHz sector probe, a linear array 6.0–13.0 MHz linear scanner and a curved array 1.8–6.0 MHz convex scanner, was used for the study. All necessary Doppler features (CD, PW, CW, and TDI) and imaging modalities (2D and M-mode) were available. For US Food and Drug Administration compliance and patient safety, the absolute maximum of the mechanical index was limited to 1.9. Images were stored digitally and analyzed immediately after each examination.

#### Ocular ultrasound

Optic nerve sheath diameter (ONSD) was measured 3 mm behind the globe with a linear probe through the closed eyelid of the patient in a supine or slightly elevated upper body (up to 20°) position. Ocular ultrasound was only performed in comatose or sedated patients. ONSD ≥ 5.2 mm was regarded as indicative of intracranial pressure (ICP) above 20 mmHg [11]. More details are given in Additional file 16.

#### Vascular ultrasound

A linear probe (veins) and a convex probe (inferior vena cava (IVC) and aorta) were used for assessments. Thrombosis of the internal jugular veins, axillary veins, femoral veins and popliteal veins was primarily evaluated by compression sonography [12–14] on one or multiple sites of the vessel. For the assessment of proximal deep vein thrombosis, the three-point compression ultrasonography method was employed. Traditional radiographic ultrasound assessments were only performed in cases of obscure or non-distinctive findings. Patients were positioned supine or with the upper body elevated. To assess the lower extremity, the leg was externally rotated and the knee flexed. The IVC was followed from the right atrium until no longer visible. In assessing hypovolemia, diagnostic criteria differed between spontaneously breathing [15] and ventilated patients [16]. The abdominal aorta was reviewed from the epigastrium to the aorto-iliac junction. Aortic diameter ≥ 3 cm was classified as aneurysmal [17].

#### Pulmonary ultrasound

A linear probe with most filters disabled in a manufacturer-defined lung preset was used for lung ultrasonography. The examination was performed according to evidence-based recommendations [18] and previously described methods [19, 20]. Four left and four right chest zones were routinely evaluated: upper anterior, lower anterior, upper lateral and basolateral. Other zones were only evaluated when necessary. Sonographic abnormalities such as interstitial syndrome (presence of B-lines ± echocardiographic abnormalities), lung consolidation (tissue-like echotexture with loss of lung aeration), pneumothorax (A-lines, absent lung sliding, presence of lung point), pulmonary edema (bilateral B-pattern and absence of other causes of B-pattern + echocardiographic abnormalities), pneumonic infiltrates (A/B-pattern, absence of lung sliding, dynamic air bronchograms), pleural effusion ((PLE); anechoic echotexture, presence of quad sign, sinusoid sign or lung line), atelectasis (consolidation plus lung sliding abolition, static air bronchograms) and compression atelectasis (atelectasis with partial re-aeration on inspiration in the presence of PLE) were assessed. (Sonographic signs for pathologic states are only given as examples, and the list is not exhaustive.) The diagnoses of interstitial syndrome and pulmonary edema were made in conjunction with other pulmonary and echocardiographic examination results according to new evidence [21] and therefore deviate from current recommendations.

#### Cardiac ultrasound

Echocardiography was performed at standardized echocardiographic windows [22] according to international guidelines [23, 24] using a sector scanner in the left lateral, supine or elevated upper body position. Particular abnormalities requiring further evaluation were examined by advanced echocardiography [25, 26]. As part of a WBU concept, the recommendations had to be slightly adjusted. Left ventricular ejection fraction (LVEF) was primarily assessed visually; Simpson’s method was used in non-distinctive cases. Heart valve disease was primarily assessed using rapid methods (e.g., vena contracta measurements, Doppler assessment) and only progressed to more elaborate and detailed methods (e.g., proximal isovelocity surface area (PISA)) if the findings were inconclusive. Right ventricular function [15] (measured by tricuspid annular plane systolic excursion) and systolic pulmonary artery pressure (sPAP) were only measured if right ventricular function was visibly impaired. These were reported as additional findings. Intracardiac hypovolemia was diagnosed when ventricular collapse, the papillary muscle kissing sign or more elaborate signs such as very small end-diastolic areas (< 8 cm^2^) and velocity time integral (VTI) variations in the left ventricular outflow tract were present [27]. Details on the assessment of hypovolemia are given in Additional file 7. The presence and location of any pericardial effusion (PE) and the diastolic width were reported. Hemodynamic compromise was evidenced by right atrial or ventricular diastolic collapse and distinctive pulsed wave Doppler transvalvular (mitral or tricuspid valve) velocities during the respiratory cycle [25, 28].

#### Abdominal ultrasound

A convex probe was primarily used for the examination, although a linear scanner was used to further evaluate specific conditions such as liver cirrhosis or pneumoperitoneum. Abdominal ultrasound examinations were usually conducted in a supine or, rarely, in an elevated upper body position (if medically indicated, for example if the ICP were raised). All four abdominal quadrants were assessed. Appropriate probe positions were chosen to evaluate the gallbladder, pancreas, liver, spleen, kidneys, and gastrointestinal and urinary tracts. Sonographic evaluation, primarily focused on predefined abnormalities, was performed according to established methods [29–32] or standard signs and measurements. Signs of pancreatitis were rated as positive when one of the following signs was present: calcifications, atrophic/fibrotic parenchyma, diffuse enlargement/volume increase, duct dilatation, presence of pseudocysts, presence of pseudoaneurysms, necrotic hypoechoic regions, focal masses, hypoechoic peripancreatic areas of inflammation/acute peripancreatic free fluid, decreased pancreatic echogenicity, or evidence of biliary obstruction. Suspected cholecystitis was rated as positive when one of the following signs was strongly positive or two signs were present: Murphy sign in awake patients, gallbladder wall thickening above 3 mm, gallstone obstruction, distension of the gallbladder lumen, sloughed intraluminal membranes, pericholecystic free fluid, intraluminal or intramural air, or hyperemic wall in (color or power) Doppler study.

In addition to evaluating predefined abnormalities, extended visual attention was paid to non-predefined signs; however, solid organs such as the liver were not evaluated for masses or other abnormalities in detail, and therefore not every single segment of the liver was screened.

If available, discharge letters and patient records were reviewed for preexisting sonographically detectable abnormalities after performing the first sonographic assessment. Sonographic findings were compared to known abnormalities and rated as (1) reproducible/plausible, if abnormalities were detected on the acquired images, or, if not detectable, their earlier presence was possible; or (2) non-reproducible/not plausible, if known abnormalities could not be reproduced even though imaging quality was acceptable, or if the presence of the pathology is or was highly unlikely (e.g., ischemic heart disease with an LVEF previously known to be severely abnormal but now appears normal LVEF despite no intervention).

Sonographic image quality was rated taking into account the well-known ICU limitations such as mechanical ventilation and restricted patient positioning using a novel rating classification in which a score for each organ system and every examination was assigned: 1 = optimal; 2 = good; 3 = sufficient; 4 = substandard; 5 = partially insufficient; 6 = mostly insufficient; 7 = assessment not possible.

A detailed written report was issued immediately after each examination, and the sonographic findings were presented to the attending intensivist, who had access to all obtained images and who assessed the images in combination with the patient’s clinical status. The attending intensivist then had to rate the clinical value of the sonographic findings on a scale of one to ten and determine whether the patient’s therapy was to be altered as a direct result of the assessment. Every intervention or adjustment that resulted solely from the sonographic assessment, such as changes in pharmacological therapy, invasive interventions or alterations in ventilator settings, was considered as therapy changes. Implementation of therapy changes was performed on the same day as the ultrasound assessment.

### Data handling and statistical analysis

Sonographic findings, intensivist ratings, patient characteristics and other relevant data from patient records were entered into a database manually. They were then pseudonymized, digitized and processed further with Microsoft Excel (USA, Redmond, WA). Basic statistical parameters were computed with Microsoft Excel. SigmaPlot (version 12.5, Systat Software Inc., USA, San Jose, CA) and OriginPro (version 9.2, OriginLab Corporation, USA, Northampton, MA) were used for more elaborate statistics. Data were compared using the *t* test for independent data and one-way analysis of variance (Holm–Šidák post hoc testing) for multiple group comparisons. Assessment of associations and correlations was evaluated by regression analysis. A *p* value of less than 0.05 was considered statistically significant. Unless stated otherwise, all continuous and categorical variables refer to examinations.

## Results

### Patient characteristics and mortality

Detailed patient characteristics including Simplified Acute Physiology Scores II (SAPS II), catecholamine therapy, mechanical ventilation status and patient categories are shown in Additional file 2. Mortality predicted by SAPS II was 23.7%, while the actual mortality was 10.8%.

### Sonographic findings and previously documented sonographic abnormalities

**Fig. 1:**
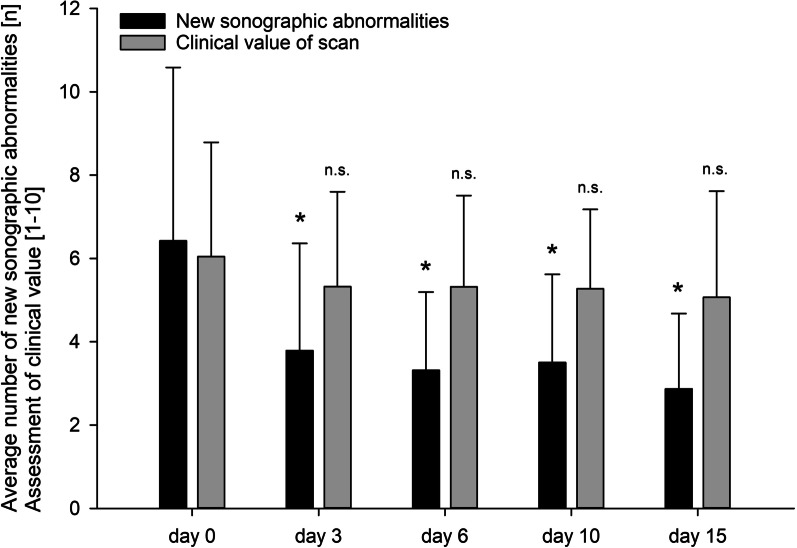
Incidence of new sonographic abnormalities, and assessment of clinical value of scan as rated by the attending intensivist, by day of scan. Data are expressed as means. Error bars show standard deviation. A detailed written report was issued immediately after each examination, and the sonographic findings were presented to the attending intensivist and put into perspective of the patient’s clinical status. The attending intensivist then had to rate the clinical value of the sonographic findings on a scale of one to ten and determine whether the patient’s therapy was to be altered as a direct result of the assessment. Asterisks = statistically significant difference (*p* < 0.05)

Two hundred and fifty-five routine sonographic assessments were performed on 111 patients on the day of admission and on days 3, 6, 10 and 15 of the intensive care stay, comprising assessments of 1275 individual organ systems and 4395 individual single-organ examinations. A total of 2484 predefined abnormalities and 519 non-predefined ICU-relevant abnormalities were detected (Additional file 3). The number of newly detected sonographic abnormalities ranged from a mean of 6.4 ± 4.2 on admission to a mean of 2.9 ± 1.8 on day 15 (Fig. 1).

In 64.9% of the patients, diagnoses of sonographically detectable abnormalities were found in previous discharge letters and patient records. These diagnoses were reproducible in 83.3%. However, only 6.9% of the patients with sonographically detected abnormalities in this study had only these abnormalities in isolation, while 93.1% of the patients exhibited additional abnormalities, not previously reported (Additional file 4).

### Grading of sonographic imaging quality

**Fig. 2:**
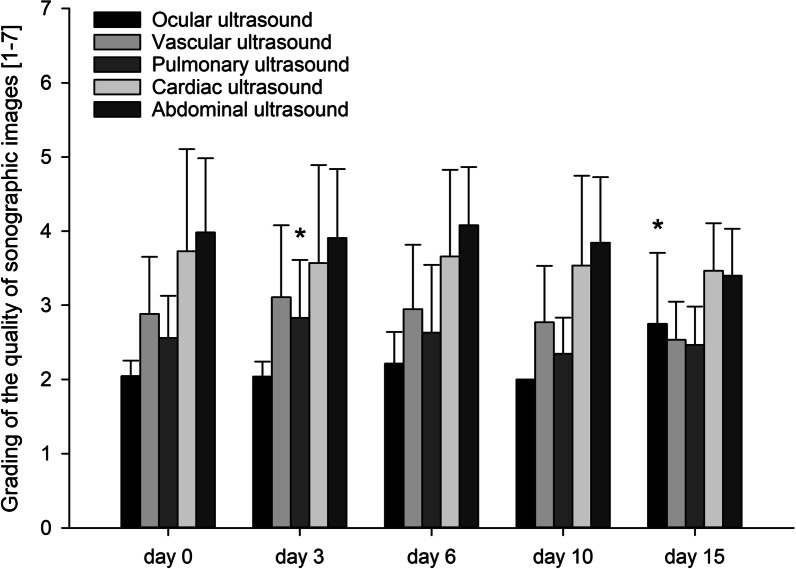
Grading of the quality of sonographic images, by day of scan. Data are expressed as means. Error bars show standard deviation. A score for each organ system and every examination was assigned: 1 = optimal; 2 = good; 3 = sufficient; 4 = substandard; 5 = partially insufficient; 6 = mostly insufficient; 7 = assessment not possible. Asterisks = statistically significant difference (between the same body part on different days; *p* < 0.05)

Longitudinal imaging quality ratings are shown in Fig. 2. Of the ocular assessments, 98.9% received a score of ≤ 3 (sufficient or better), followed by pulmonary (95.7%) and vascular assessments (88.6%). Cardiac and abdominal assessments showed lower values (49.4% and 34.9%, respectively).

### Ocular ultrasound

ICP over 20 mmHg was suspected in 3.1% of all examinations in three patients (Additional file 3). Suspected increased ICP was confirmed by intracranial microtransducer measurements in two patients. In one of these patients, the clinical presentation was consistent with the sonographic findings and increased ICP was treated without invasive confirmation.

### Vascular ultrasound

The incidence of thrombosis increased significantly from 16.2% on admission to 60% on day 15 (Additional file 5). The IVC showed signs of volume deficit in 25.9% and was distended in 38%. Abdominal aortic aneurysms were detected in 2%; however, ICU-specific limitations of image quality restricted assessments of the entire abdominal aorta in the majority of patients. Detailed results and non-predefined abnormalities are represented in Additional file 3.

### Pulmonary ultrasound

**Fig. 3:**
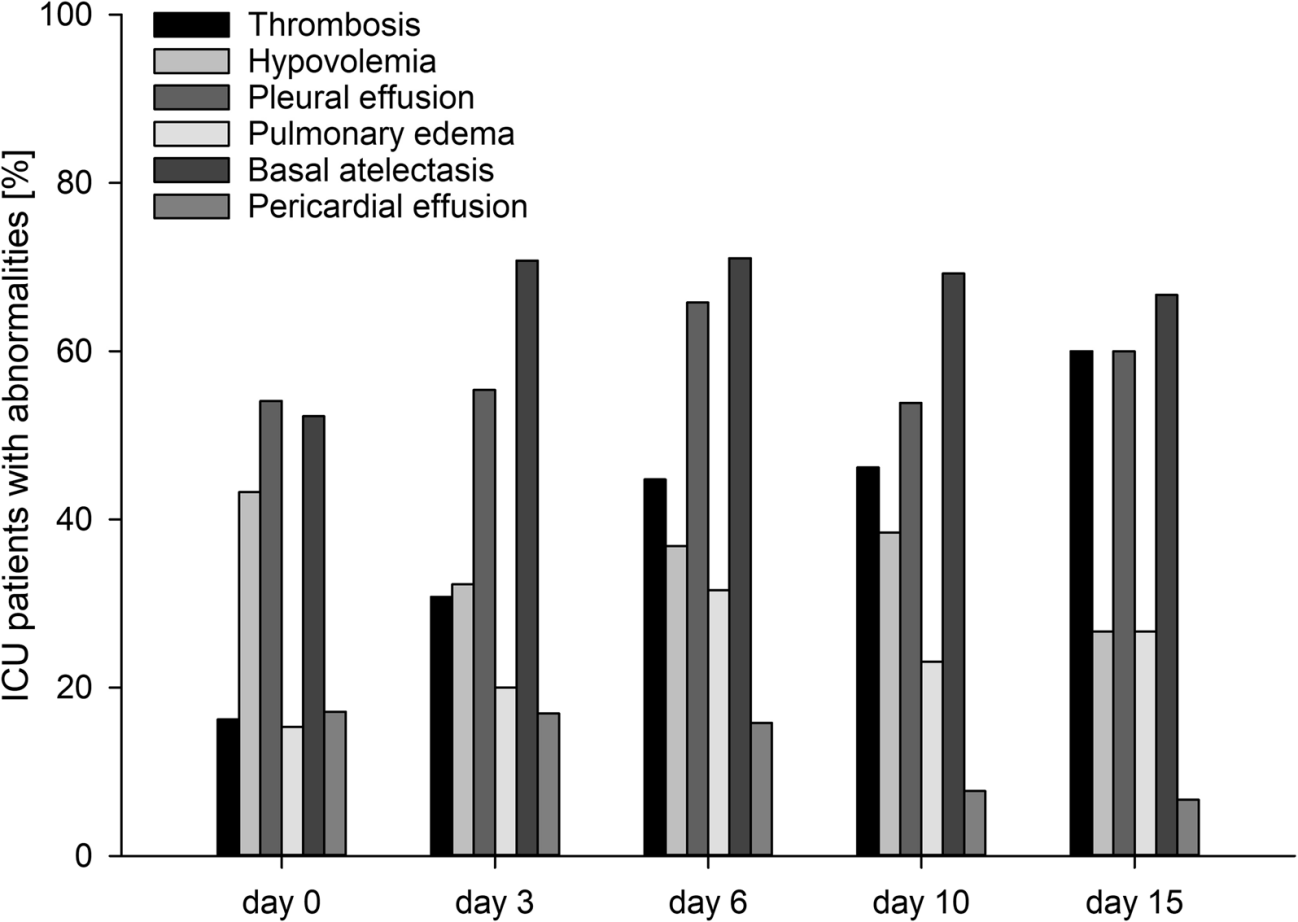
Incidence of common pulmonary sonographic abnormalities and hypovolemia detected using the FASP-ICU protocol, by day of scan

Interstitial syndrome was present in 26.7%, lung consolidations in 75.7%, and signs of pneumothorax in 12.9% of all examinations. Interstitial syndrome could be attributed to pulmonary edema in 76.5% of scans where interstitial syndrome was present (20.4% of all examinations); lung consolidations could be attributed to basal atelectasis in 70.3% (62.4% of all examinations), and signs of pneumothorax proved to be pneumothorax by the detection of the lung point (3.5% of all examinations) in 21.1% for the left lung and in 40% for the right lung. PLEs were detected in 56.5%, for which thoracocentesis was indicated in 23.2% of left-sided and in 16.8% of right-sided PLEs. Of the cases of basal atelectasis, 20.9% were due to compression atelectasis. Detailed results and non-predefined abnormalities are represented in Additional file 3, and partial results in Fig. 3 and Additional file 6.

### Echocardiography

LVEF was normal in 68.2%, mildly abnormal in 9.4%, moderately abnormal in 8.2% and severely abnormal in 5.9% of all examinations. Pericardial effusions were present in 15.3% of which none was hemodynamically significant. Clinically significant heart valve disease was defined as moderate or severe heart valve regurgitation or stenosis. Relevant regurgitation was detected in 13.3%, and stenosis in 4.3%. Signs of volume deficit were measured in 18.4% for the left ventricle, and in 14.9% for the right ventricle. Taking together the results of vascular and cardiac assessments, 43.2% of the patients had signs of intravascular or intracardiac hypovolemia on admission, and the incidence was still 38.4% on day 10 (Additional file 7). Detailed results and non-predefined abnormalities are presented in Additional file 3, and partial results in Fig. 3 and Additional files 8 and 9.

### Focused abdominal ultrasound

**Fig. 4:**
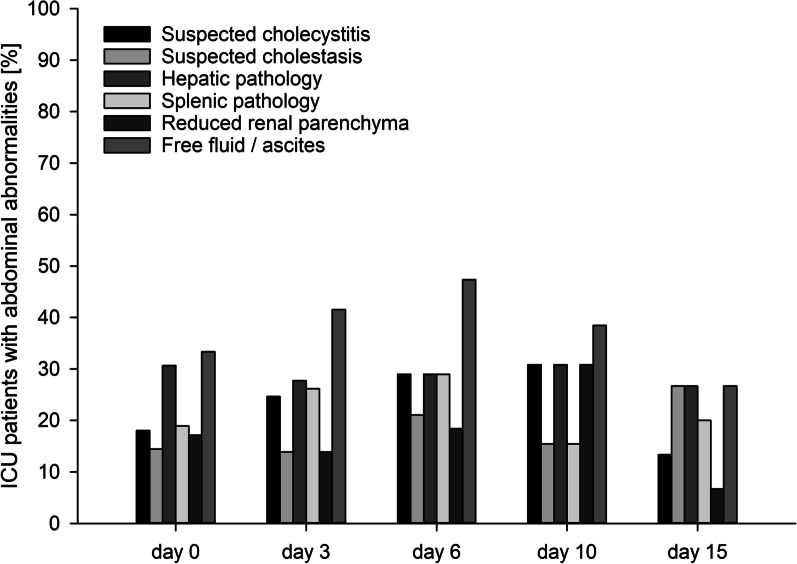
Incidence of common abdominal abnormalities detected using the FASP-ICU protocol, by day of scan

Sonographic signs suggestive of cholecystitis or pancreatitis were found in 22.4% and 1.6% of the examinations, respectively. Overall, 29.4% of the scans revealed liver abnormalities such as hepatomegaly (15.7%), signs of cirrhosis (4.3%), or dilated portal (4.3%) or hepatic veins (8.6%). Ultrasound of the spleen revealed abnormalities in 22%. Renal atrophy (6.7%), hypertrophy (22.4%) and reduced renal parenchyma (16.9%) were common kidney abnormalities, while hydronephrosis (1.2%) and urolithiasis (0%) were rare or absent. Assessment of intestinal motility showed that peristalsis was normal in 34.5%, reduced in 57.3% and absent in 6.3% with 3.1% of the examinations revealing sonographic signs of ileus. Pneumoperitoneum was detected in 5.5% of routine ultrasound examinations (mainly due to interventions or operations). Minimal amounts of free abdominal fluid were seen in 19.2%, moderate in 16.1% and massive in 2.4% of the examinations. Detailed results and additional non-predefined abnormalities are presented in Additional file 3, and partial results in Fig. 4 and Additional file 10–14.

### Value scale scores, examination time and changes to therapy

The clinical value of the sonographic findings was rated by the attending intensivists. The mean scores were 6.0 ± 2.7 for the initial examination and 5.1 ± 2.5 for the final examination on day 15 (Fig. 1). Newly detected abnormalities in ultrasonographic examinations directly led to changes in the therapeutic regimen after 45.1% of all examinations, with changes in respirator settings (frequent PEEP level adjustments), adjustments of fluid therapy in response to evidence of hypovolemia, hypervolemia and interstitial syndrome, and pharmacotherapy (especially anticoagulants, antibiotics and opioids) being common. The assessment on day 3 was followed by the lowest rate of therapy changes (40%), while the final assessment was followed by the highest rate of therapy changes (60%, Table 1). The mean total examination time was 18.7 ± 3.2 min (range 8.3–31.2 min), resulting in 1.6 min invested per detected pathology. The proportion of examinations completed within 20 min was 68.5%, and 87.9% were completed within 22 min. Mean examination times are shown in Table 1.

**Table 1:** Incidence of changes to therapy, and mean examination times (pure scanning times without reporting), by day of scan

	Incidence of therapy changes due to FASP (%)	Mean examination time (mm:ss)
Day 0	46.8	19:12
Day 3	40.0	17:43
Day 6	44.7	19:04
Day 10	42.3	18:16
Day 15	60.0	19:10
Overall	45.1	18:40

### Correlations between ICU parameters and sonographic values

SAPS II, number of days in intensive care and patient age underwent regression analysis in order to evaluate their predictive value with regards to new sonographic findings, the value scale score and therapy changes. No clinically useful correlations were identified (Additional file 15).

## Discussion

By evaluating repeat surveillance ultrasound examinations of ICU patients over the course of 15 days following a routine WBU protocol, all predefined study aims were met. The range and the prevalence of common abnormalities were defined, and the protocol proved to be useful, practicable and feasible within the existing time constraints. The WBU protocol demonstrated its value as a routine examination tool by detecting abnormalities before they became clinically evident or could result in serious adverse events. Accordingly, the actual mortality rate of study participants was more than 50% lower than that predicted by the SAPS II, although the actual and the SAPS II predicted mortality rates are generally within the same range in our institution. It further showed its clinical value by receiving high ratings by the intensivists in charge. The results obtained by following the WBU protocol revealed the necessity of changing therapeutic regimens in 45.1% of the cases.

Despite the positive evaluation of the FASP-ICU protocol, the range of pathologic findings clearly defined it as a non-exhaustive screening tool. The predefined abnormalities relevant to intensivists exceeded the non-listed abnormalities detected as additional findings. However, some very significant abnormalities such as occlusion of the portal vein are not part of the protocol because they are difficult to detect and therefore not yet practicable for an intensivist’s ultrasound skills.

With 3003 detected sonographic abnormalities including 2484 predefined abnormalities, intensivists were able to adjust therapy early, before conditions deteriorated and potentially caused complications. The large number of affected organ systems evidently calls for WBU examinations instead of only assessing single or clinically already compromised organ systems as is the current practice. Interestingly but not surprisingly, the abnormalities were not static but highly dynamic as shown by the newly detected abnormalities on each serial examination. Taking into account the results of the value scale score of the intensivist in charge, which did not significantly vary for serial examinations, we recommend using WBU on a routine basis every 3 to 5 days of ICU treatment (unless deterioration of vital systems requires additional urgent focused ultrasonographic assessments).

The protocol was completed within 22 min in almost 90% of patients. In view of the resulting 45.1% in therapy changes, we consider the value of the protocol compared with the time required for the examination compelling.

Sonographic abnormalities did not correlate with ICU parameters such as SAPS II, indicating that all patients should be evaluated by WBU. No patient cohort was identified as benefiting the most. However, screening patient records for sonographic abnormalities revealed that 93.1% of patients with known sonographically detectable abnormalities had additional yet unknown abnormalities relevant to their ICU treatment. We therefore recommend using the WBU approach for every ICU patient or at the very least for every patient that has previously known disease states that are sonographically detectable.

Ultrasound assessments in the ICU performed by intensivists are increasing, and recommendations for training and implementation were recently issued for critical care echocardiography [23, 26, 33, 34] and ultrasonography of other organ systems [2, 4, 33, 35]. In our study, the relatively high rate of misdiagnosed findings calls for more and further detailed recommendations for these techniques. As these incorrect diagnoses were made by non-intensivist physicians, who probably perform ultrasound examinations on a more regular basis, the rate of inadequate findings produced by intensivists with less experience might be even higher. Training schemes and courses should be implemented, in line with curriculum requirements, to increase the quality of examinations and to educate a greater number of intensivists in these techniques.

The grading of imaging quality must be interpreted with knowledge of several limitations in image acquisition in the ICU (for example mechanical ventilation, restricted patient positioning, edema and inability to follow breathing commands). Within the time constraints of a WBU protocol, ocular, vascular and pulmonary assessments showed an image quality adequate for definitive interpretations, while cardiac assessments were only adequate in about half of the examinations and abdominal assessments in only about one third. However, despite the poorer image quality, the ability of an expert examiner to diagnose important abnormalities was only affected in few cases, but this might still be an important problem for operators with less experience.

The high prevalence of thrombosis (up to 60%), despite the prophylactic routine administration of heparin, is highlighted. The internal jugular veins, which were primarily used for placement of central venous catheters, were affected most frequently. ICP assessments showed a low prevalence of ICP elevation. However, ultrasonography detected two patients with increased ICP that were not suspected from the clinical presentation. The number of examinations needed to detect one unexpected ICP elevation was 33, which is a sound investment considering the immense consequences. Recent evidence evaluating the sonographic assessment of optic disc elevation as a sign for papilledema [36], in addition to ONSD, might further increase the accuracy of ultrasound for noninvasively diagnosing ICP elevation in the ICU.

Lung ultrasonography revealed a high prevalence of basal atelectasis (70.3%) despite the use of liberal positive end-expiratory pressure (PEEP) in ventilated patients and at least three cycles of non-invasive continuous positive airway pressure (CPAP) mask ventilation per day to prevent atelectasis in most spontaneously breathing patients at risk for pneumonia or atelectasis. Likewise, the high prevalence of PLEs (56.5%) and pulmonary edema (20.4%) despite concurrent intravascular or intracardiac hypovolemia (up to 43.2%; not associated with fluid responsiveness [37]) remains a management challenge.

LVEF was reduced in 23.5% of the examinations and should be taken into account in the management of these patients. While a pericardial effusion was frequently seen, it was not hemodynamically significant in any patient.

Signs of cholecystitis were also frequent, but even though a course of antibiotics was only prescribed for a minority of these patients, none progressed to sepsis or required surgical intervention.

At present, no reliable outcome data for sonographic techniques in the ICU are available. Learning from the mistakes of the past (e.g., minimal training and flawed interpretation of pulmonary artery catheter measurements), adequate training of intensivists in ICU ultrasound techniques and ideally international consensus [4] should precede any attempt to measure relevant outcome variables at this point. This might only be achieved in collaborative multicenter studies with a large number of intensivists thoroughly trained and experienced in these methods.

### Limitations of the study

Several limitations apply to our single expert operator, single-center approach. However, adjustments were made to limit possible bias. We highlight that the study results are highly dependent on the type of ICU patients and on basic patient factors.

Experienced WBU examiners are rare, and only an operator trained in internal medicine and cardiology as well as in anesthesiology and intensive care would be able to develop, execute and evaluate the original study protocol; although in its evaluated and revised form, it is intended for all intensivists working with POCUS in the ICU. Regarding our observed drop in ICU mortality, the study results cannot be used to show definitively that WBU affects outcome. Common sense favors this conclusion; however, due to the study design it remains an untested hypothesis.

The early recording of therapy changes shortly after issuing the WBU reports probably lead to an underestimation of reported therapy changes. The WBU reports frequently triggered further team discussions that led to additional therapy changes that according to the protocol were not recorded in the study.

In the evolving field of ultrasonography, there is continuous improvement in clinical practice. To acknowledge a few of these developments, amendments to our protocol have already been made, to be used in future studies, for instance by adding papilledema [36] to the ocular ultrasound protocol and updating the reference and cutoff values for LVEF (Additional file 16).

## Conclusions

By developing a feasible and rapid bedside WBU protocol to guide the assessment of 99 predefined sonographic abnormalities relevant in ICU patients, we obtained sonographic results that led experienced attending intensivists to alter their therapy regimen after 45.1% of the examinations. The detected sonographic abnormalities showed a high rate of change during the serial assessments indicating the value of routine surveillance sonographic examinations.

## Supplementary Information

**Additional file 1**. Focused assessment of sonographic abnormalities in the intensive care unit (FASP-ICU) protocol.

**Additional file 2**. Patient characteristics, catecholamine therapy, ethnicity and reason for admission of ICU patients. Continuous variables are expressed as median (range) or mean ± standard deviation and categorical variables as percentages.

**Additional file 3**. Results of FASP-ICU examinations 1–5.

**Additional file 4**. Sonographically assessable abnormalities mentioned in patients’ medical records.

**Additional file 5**. Incidence of thrombosis detected using the FASP-ICU protocol.

**Additional file 6**. Proportion of patients with pulmonary sonographic abnormalities detected using the FASP-ICU protocol.

**Additional file 7**. Proportion of patients with hypovolemia detected using the FASP-ICU protocol. Hypovolemia was assessed according to three criteria: 1 – inferior vena cava (IVC) assessment (respiratory variations in IVC diameter); 2 – right ventricular dimensions and flow measurements; 3 – left ventricular dimensions and flow measurements (intracardiac hypovolemia was diagnosed when ventricular collapse, the papillary muscle kissing sign or more elaborate signs, such as very small end-diastolic areas and velocity time integral (VTI) variations in the left ventricular outflow tract, were present). In the assessment of hypovolemia, single values should be used with care. They are assumed to have low sensitivities and specificities for diagnosing hypovolemia in ICU patients, especially single IVC assessments, which have a number of confounders and limitations. As there are no validated ultrasound criteria for hypovolemia in ICU patients, we recommend using a combination of ultrasound and non-ultrasound assessments until further evidence becomes available.

**Additional file 8**. Assessment of left ventricular ejection fraction (LVEF) by day of scan.

**Additional file 9**. Presence of heart valve abnormalities detected using the FASP-ICU protocol.

**Additional file 10**. Presence of common abdominal abnormalities detected using the FASP-ICU protocol.

**Additional file 11**. Presence of renal abnormalities detected using the FASP-ICU protocol.

**Additional file 12**. Presence of intra-abdominal free fluid / ascites detected using the FASP-ICU protocol.

**Additional file 13**. Presence of peristalsis detected using the FASP-ICU protocol.

**Additional file 14**. Proportion of patients showing liver abnormalities detected using the FASP-ICU protocol.

**Additional file 15**. Correlations between commonly used ICU parameters and sonographic values.

**Additional file 16**. Suggestions of protocol additions and alterations that were identified during the review process. They will be evaluated for inclusion in an updated FASP-ICU protocol and should be considered when executing the original version of the FASP-ICU protocol.

## Data Availability

All data generated or analyzed during this study are included in this published article and its supplementary information files except the original ultrasound images. These original ultrasound image files (> 1 TB of compressed data) are available from the corresponding author on reasonable request.

## Abbreviations

CPAP: Continuous positive airway pressure
ED: Emergency department
FASP-ICU: Focused assessment of sonographic abnormalities in the intensive care unit
IVC: Inferior vena cava
ICU: Intensive care unit
ICP: Intracranial pressure
LVEF: Left ventricular ejection fraction
ONSD: Optic nerve sheath diameter
PE: Pericardial effusion
PLE: Pleural effusion
PEEP: Positive end-expiratory pressure
PISA: Proximal isovelocity surface area
POCUS: Point-of-care ultrasound
SAPS II: Simplified Acute Physiology Score
sPAP: Systolic pulmonary artery pressure
VTI: Velocity time integral
WBU: Whole-body ultrasound
