# Supplementary material for: Development and evaluation of the focused assessment of sonographic pathologies in the intensive care unit (FASP-ICU) protocol
Source: Crit Care. 2021 Nov 24;25:405. doi: 10.1186/s13054-021-03811-2 (PMC8611927; doi:10.1186/s13054-021-03811-2)
Supplement: Supplementary file 1 — Additional file 1. Focused assessment of sonographic abnormalities in the intensive care unit (FASP-ICU) protocol. [file 13054_2021_3811_MOESM1_ESM.pdf]

University Hospital Goettingen
Department of Anesthesiology, Emergency and Intensive Care Medicine
Robert-Koch-Str. 40
37075 Goettingen/Germany

Diagnostic report FASP-ICU study

Date:
Version: 2.6.8
Initial examination: ☐
Follow-up examination: No. ____
Investigator:

Number of pseudonymization:

Patient label

Ocular ultrasound ☐ not performed / evaluation not possible ☐

☐ Optic nerve sheath diameter indicating intracerebral pressure below 20 mmHg
☐ Optic nerve sheath diameter indicating intracerebral pressure above 20 mmHg

Additional findings

Vascular ultrasound ☐ not performed / evaluation not possible ☐

Left internal jugular vein	☐ non-compressible	☐ compressible
Right internal jugular vein	☐ non-compressible	☐ compressible
Left axillary vein	☐ non-compressible	☐ compressible
Right axillary vein	☐ non-compressible	☐ compressible
Left femoral vein	☐ non-compressible	☐ compressible
Right femoral vein	☐ non-compressible	☐ compressible
Left popliteal vein	☐ non-compressible	☐ compressible
Right popliteal vein	☐ non-compressible	☐ compressible

Inferior vena cava
☐ not performed / evaluation not possible
☐ distended IVC (normal with PEEP)
☐ non-distended IVC
☐ IVC indicating volume deficit

Abdominal aorta
☐ not assessed / examination not possible
☐ no aneurysm
☐ aneurysm _Ø_________ cm
☐ aorta not assessable in entire length; no aneurysm in assessable sections

Additional findings

Pulmonary ultrasound

☐ not performed / evaluation not possible ☐

Physiologic lung sliding	☐ yes	☐ absent on left	☐ absent on right	
Interstitial syndrome (B-pattern)	☐ no	☐ left	☐ right	☐ bilateral
Lung consolidation	☐ no			
	☐ left	☐ apical	☐ medial	☐ basal
	☐ right	☐ apical	☐ medial	☐ basal
Signs of pneumothorax	☐ no	☐ left	☐ right	
Pneumothorax (detection of lung point)	☐ no	☐ left	☐ right	
Pulmonary edema	☐ no	☐ yes		
Suspected pneumonic infiltrates	☐ no	☐ left	☐ right	
Pleural effusion / hemothorax	☐ no	☐ left	☐ right	
Thoracentesis indicated	☐ no	☐ left	☐ right	
Basal atelectasis	☐ no	☐ left	☐ right	
Compression atelectasis	☐ no	☐ left	☐ right	

Additional findings

Focused echocardiography

☐ not performed / evaluation not possible ☐

Visual left ventricular ejection fraction (LVEF): ☐ not performed / evaluation not possible

☐ normal (LVEF > 55%)

☐ mildly abnormal (LVEF > 45–55%)

☐ moderately abnormal (LVEF 30–45%)

☐ severely abnormal (LVEF < 30%)

☐ segmental wall motion abnormalities ☐ global hypokinesia / akinesia

Pericardial effusion: ☐ not performed / evaluation not possible

☐ no ☐ yes, not hemodynamically significant ☐ yes, hemodynamically significant

Heart valve disease: ☐ not performed / evaluation not possible

Moderate to severe heart valve regurgitation

☐ no ☐ aortic valve ☐ mitral valve ☐ tricuspid valve

Moderate to severe heart valve stenosis

☐ no ☐ aortic valve ☐ mitral valve ☐ tricuspid valve

Echocardiographic signs of volume deficit: ☐ not performed / evaluation not possible

☐ no ☐ yes, left ventricle ☐ yes, right ventricle

Additional findings

Focused abdominal ultrasound ☐ not performed / evaluation not possible ☐

Gallbladder	☐ not performed / evaluation not possible			
	☐ no obvious abnormalities			
	☐ gallbladder not sufficiently assessable	☐ sludge		
	☐ cholelithiasis	☐ atonic gallbladder		
Suspected cholecystitis	☐ no	☐ wall thickening > 3 mm	☐ perivesicular fluid	
Suspected cholestasis	☐ no	☐ dilated CBD _____ mm	☐ double-barrel phenomenon	
Pancreas	☐ not performed / evaluation not possible			
	☐ no obvious abnormalities			
	☐ signs of pancreatitis			
Liver	☐ not performed / evaluation not possible			
	☐ no obvious abnormalities			
	☐ reduced liver size		☐ liver enlargement (hepatomegaly)	
	☐ signs of cirrhosis		☐ dilated hepatic portal vein	
	☐ dilated hepatic veins		☐ suspected liver hematoma	
Spleen	☐ not performed / evaluation not possible			
	☐ no obvious abnormalities			
	☐ splenomegaly		☐ splenic hematoma	
Kidneys	☐ not performed / evaluation not possible			
Kidney size	☐ normal			
	☐ reduced	☐ left	☐ right	
	☐ enlarged	☐ left	☐ right	
Dilated renal pelvis	☐ no	☐ left	☐ right	
Reduced renal parenchyma	☐ no	☐ yes		
Urolithiasis	☐ no	☐ yes	Location: _______________	
Intestinal	☐ not performed / evaluation not possible			
Peristalsis	☐ normal	☐ weak	☐ absent	
Suspected ileus	☐ no	☐ yes		
Urinary bladder	☐ not performed / evaluation not possible			
	☐ empty	☐ filled	☐ distended	☐ bladder tamponade
Pneumoperitoneum	☐ not performed / evaluation not possible			
	☐ no	☐ yes		
Free fluid / ascites	☐ not performed / evaluation not possible			
	☐ no	☐ minimal	☐ moderate	☐ massive
Location	☐ diffuse	☐ hepatorenal recess		

☐ splenorenal recess ☐ around bladder
☐ other location: ______________________________

Additional findings

Legend

Sonographic conditions (quality rating) ☐

Sonographic assessment quality grading as rated in the context of ICU limitations:

1 = optimal 2 = good 3 = sufficient 4 = substandard 5 = partially insufficient
6 = mostly insufficient 7 = assessment not possible

Mark option ☒ or ☑
