# Supplementary material for: Development and evaluation of the focused assessment of sonographic pathologies in the intensive care unit (FASP-ICU) protocol
Source: Crit Care. 2021 Nov 24;25:405. doi: 10.1186/s13054-021-03811-2 (PMC8611927; doi:10.1186/s13054-021-03811-2)
Supplement: Supplementary file 2 — Additional file 2. Patient characteristics, catecholamine therapy, ethnicity and reason for admission of ICU patients. Continuous variables are expressed as median (range) or mean ± standard deviation and categorical variables as percentages. [file 13054_2021_3811_MOESM2_ESM.pdf]

Additional file 2 Patient characteristics, catecholamine therapy, ethnicity, and reason for admission of ICU patients

**Gender**	male	*64 (n) 57.7 (%)*	female	*47 (n) 42.3 (%)*
**Age [years]**	mean	*68.7 ± 12.9*	median	*72 (24–90)*
**Body weight [kilograms]**	mean	*79.7 ± 17.3*	median	*77 (36–150)*
**Body mass index [kg·m^{-2}]**	mean	*27.0 ± 5.1*	median	*26.3 (14.1–55.1)*
	>30	*24 (n) 21.6 (%)*	>40	*2 (n) 1.8 (%)*

**Days in ICU**		
mean	*6.5 ± 8.0*	
median	*3 (1–36)*	
**Survival rate**		
survived	*99 (n) 89.2 (%)*	
died	*12 (n) 10.8 (%)*	

**SAPS II score**	mean	median
**day 0**	*39.4 ± 12.8*	*40 (6–67)*
**day 3**	*38.4 ± 11.8*	*39 (13–64)*
**day 6**	*41.7 ± 9.1*	*41 (27–60)*
**day 10**	*42 ± 9.3*	*41.5 (27–66)*
**day 15**	*34.5 ± 7.5*	*34 (24–45)*

**Mechanically ventilated?**			**Catecholamine therapy?**	
	ventilated	spontaneous	yes	no
**day 0**	*62 (n) 55.9 (%)*	*49 (n) 44.1 (%)*	*49 (n) 44.1 (%)*	*62 (n) 55.9 (%)*
**day 3**	*32 (n) 49.2 (%)*	*33 (n) 50.8 (%)*	*19 (n) 29.2 (%)*	*46 (n) 70.8 (%)*
**day 6**	*20 (n) 52.6 (%)*	*18 (n) 47.4 (%)*	*9 (n) 23.7 (%)*	*29 (n) 76.3 (%)*
**day 10**	*11 (n) 42.3 (%)*	*15 (n) 57.7 (%)*	*4 (n) 15.4 (%)*	*22 (n) 84.6 (%)*
**day 15**	*7 (n) 46.7 (%)*	*8 (n) 53.3 (%)*	*2 (n) 13.3 (%)*	*13 (n) 86.7 (%)*

**Ethnicity**	Number	%
White	198	98.2
Hispanic	1	0.9
Asian	1	0.9

Reason for admission of surgical ICU patients:

sepsis 8

pneumonia / ARDS 7

trauma 6

multiple trauma 4

thoracic surgery 6

cardiac surgery 19

intracranial bleeding	11
malignancies and complications	10
neurosurgery	10
OB/GYN surgery	3
abdominal surgery	9
cardiac	11
other (transplantation, intoxication, hemorrhagic shock, etc.)	7

Continuous variables are expressed as median (range) or mean ± standard deviation and categorical variables as percentages.
