# Supplementary material for: Development and evaluation of the focused assessment of sonographic pathologies in the intensive care unit (FASP-ICU) protocol
Source: Crit Care. 2021 Nov 24;25:405. doi: 10.1186/s13054-021-03811-2 (PMC8611927; doi:10.1186/s13054-021-03811-2)
Supplement: Supplementary file 3 — Additional file 3. Results of FASP-ICU examinations 1–5. [file 13054_2021_3811_MOESM3_ESM.pdf]

Ocular ultrasound	Not performed / evaluation not possible: 157 (65.6%)

Optic nerve sheath diameter indicating intracerebral pressure below 20 mmHg: 95 (96.9%)
Optic nerve sheath diameter indicating intracerebral pressure above 20 mmHg: 3 (3.1%)

Additional findings
None

Vascular ultrasound	Not performed / evaluation not possible: 0 (0%)

Left internal jugular vein	non-compressible: 27 (10.6%)	compressible: 222 (87.1%)
Right internal jugular vein	non-compressible: 46 (18%)	compressible: 192 (75.3%)
Left axillary vein	non-compressible: 2 (0.8%)	compressible: 246 (96.5%)
Right axillary vein	non-compressible: 0 (0%)	compressible: 251 (98.4%)
Left femoral vein	non-compressible: 7 (2.7%)	compressible: 226 (88.6%)
Right femoral vein	non-compressible: 6 (2.4%)	compressible: 225 (88.2%)
Left popliteal vein	non-compressible: 6 (2.4%)	compressible: 233 (91.4%)
Right popliteal vein	non-compressible: 10 (3.9%)	compressible: 238 (93.3%)

Inferior vena cava	not performed / evaluation not possible: 37 (14.5%)
	distended IVC (normal with PEEP): 97 (38%)
	non-distended IVC: 54 (21.2%)
	IVC indicating volume deficit: 66 (25.9%)
Abdominal aorta	not performed / evaluation not possible: 120 (47.1%)
	no aneurysm: 30 (11.8%)
	aneurysm: 5 (2%)
	aorta not assessable in full length; no aneurysm in assessable sections: 98 (38.4%)

Additional findings (non-exhaustive list)
Arteriovenous fistula after percutaneous coronary intervention

Carotid stenosis	Inferior vena cava thrombus
Arterial calcification and stenosis	Cysts and masses in thyroid gland
Abdominal / femoral stents	Acute arterial occlusion

Pulmonary ultrasound	Not performed / evaluation not possible: 98 (38.4%)

Physiologic lung sliding	yes: 203 (79.6%)	
	absent on left: 23 (9%)	absent on right: 30 (11.8%)
Interstitial syndrome (B-profile)	no: 185 (72.5%)	

	focal left: 15 (5.9%)	focal right: 11 (4.3%)	bilateral: 42 (16.5%)
Lung consolidation	no: 60 (23.5%)		
	left: 171 (67.1%)		
	apical: 1 (0.4%)	medial: 41 (16.1%)	basal: 171 (67.1%)
	right: 157 (61.6%)		
	apical: 2 (0.8%)	medial: 22 (8.6%)	basal: 156 (61.2%)
Signs of pneumothorax	no: 156 (61.2%)		
	left: 19 (7.5%)	right: 15 (5.9%)	
Pneumothorax (detection of lung point)			
	no: 244 (95.7%)		
	left: 4 (1.6%)	right: 6 (2.4%)	
Pulmonary edema	no: 202 (79.2%)	yes: 52 (20.4%)	
Suspected pneumonic infiltrates	no: 234 (91.8%)	left: 10 (3.9%)	right: 6 (2.4%)
Pleural effusion / hemothorax	no: 109 (42.7%)	left: 125 (49%)	right: 95 (37.3%)
Thoracentesis indicated	left: 29 (11.4%)	right: 16 (6.3%)	
Basal atelectasis	no: 94 (36.9%)	left: 132 (51.8%)	right: 98 (38.4%)
Compression atelectasis	no: 209 (82%)	left: 27 (10.6%)	right: 21 (8.2%)

Additional findings (non-exhaustive list)

Masses, suspected metastases	Lung contusion
Rib fractures	Suspected pleural empyema
Pulmonary emphysema	Diaphragmatic elevation
Lung resection	Subcutaneous emphysema (aerodermectasia)
Pleural effusions despite chest tube	

Focused echocardiography Not performed / evaluation not possible: 11 (4.3%)

Visual left ventricular ejection fraction (LVEF)

not performed / evaluation not possible: 10 (3.9%)
segmental wall motion abnormalities: 29 (11.4%)
global hypokinesia / akinesia: 13 (5.1%)
normal (LVEF > 55%): 174 (68.2%)
mildly abnormal (LVEF > 45–55%): 24 (9.4%)
moderately abnormal (LVEF 30–45%): 21 (8.2%)
severely abnormal (LVEF < 30%): 15 (5.9%)

Pericardial effusion
not performed / evaluation not possible: 9 (3.5%)
no: 195 (76.5%)
yes, not hemodynamically significant: 39 (15.3%)
yes, hemodynamically significant: 0 (0%)

Heart valve disease
not performed / evaluation not possible: 37 (14.5%)

Moderate to severe regurgitation

no: 173 (67.8%)
aortic valve: 10 (3.9%)
mitral valve: 12 (4.7%)
tricuspid valve: 16 (6.3%)

Moderate to severe heart valve stenosis

no: 196 (76.9%)
aortic valve: 11 (4.3%)
mitral valve: 1 (0.4%)
tricuspid valve: 0 (0%)

Echocardiographic signs of volume deficit

no: 182 (71.4%)
yes, left ventricle: 47 (18.4%)
yes, right ventricle: 38 (14.9%)

Additional findings (non-exhaustive list)

Hypertrophic cardiomyopathy with systolic anterior motion (SAM) of mitral valve leaflet

Endocarditis	Floating structure in left ventricle after cardiac surgery
Ascending aortic aneurysm	Atrial septal aneurysm
Suspected pulmonary hypertension	Right ventricular dysfunction
Floating structure in right atrium	Myocardial aneurysm
Moderate pulmonary insufficiency	Impella® pump malposition
Stenosis of mechanical mitral valve	Left ventricular air bubbles
Right ventricular dilatation	

Focused abdominal ultrasound

Not performed / evaluation not possible: 3 (1.2%)

Gallbladder	not performed / evaluation not possible: 11 (4.3%)
	no obvious abnormalities: 97 (38%)
	gallbladder not sufficiently assessable: 62 (24.3%)
	sludge: 46 (18%)
	cholelithiasis: 32 (12.5%)
	atonic gallbladder: 10 (3.9%)
Suspected cholecystitis	no: 128 (50.2%)
	wall thickening > 3 mm: 45 (17.6%)
	perivesicular fluid: 12 (4.7%)
Suspected cholestasis	no: 187 (73.3%)
	dilated CBD: 42 (16.5%)
	double-barrel phenomenon: 0 (0%)
Pancreas	not performed / evaluation not possible: 196 (76.9%)
	no obvious abnormalities: 43 (16.9%)

	signs of pancreatitis: 4 (1.6%)		
<u>Liver</u>	not performed / evaluation not possible: 4 (1.6%)		
	no obvious abnormalities: 4 (1.6%)		
	reduced liver size: 0 (0%)		
	liver enlargement (hepatomegaly): 40 (15.7%)		
	signs of cirrhosis: 11 (4.3%)		
	dilated hepatic portal vein: 11 (4.3%)		
	dilated hepatic veins: 22 (8.6%)		
	suspected liver hematoma: 1 (0.4%)		
<u>Spleen</u>	not performed / evaluation not possible: 9 (3.5%)		
	no obvious abnormalities: 187 (73.3%)		
	splenomegaly: 42 (16.5%)		
	splenic hematoma: 3 (1.2%)		
<u>Kidneys</u>	not performed / evaluation not possible: 6 (2.4%)		
Kidney size	normal: 172 (67.5%)		
	reduced: 17 (6.7%)	left: 9 (3.5%)	right: 15 (5.9%)
	enlarged: 57 (22.4%)	left 39 (15.3%)	right: 44 (17.3%)
Dilated renal pelvis	no: 235 (92.2%)	left: 5 (1.2%)	right: 2 (0.8%)
Reduced renal parenchyma			
	no: 201 (78.8%)	yes: 43 (16.9%)	
Urolithiasis	no: 243 (95.3%)	yes: 0 (0%)	
<u>Intestinal</u>	not performed / evaluation not possible: 1 (0.4%)		
Peristalsis	normal: 88 (34.5%)	weak: 146 (57.3%)	absent: 16 (6.3%)
Suspected ileus	no: 242 (94.9%)	yes: 8 (3.1%)	
<u>Urinary bladder</u>	not performed / evaluation not possible: 77 (30.2%)		
	empty: 154 (60.4%)		
	filled: 13 (5.1%)		
	distended: 6 (2.4%)		
	bladder tamponade: 0 (0%)		
<u>Pneumoperitoneum</u>	not performed / evaluation not possible: 235 (92.2%)		
	no: 3 (1.2%) yes: 14 (5.5%)		
<u>Free fluid / ascites</u>	not performed / evaluation not possible: 0 (0%)		
	no: 156 (61.2%)		
	minimal: 49 (19.2%)		
	moderate: 41 (16.1%)		
	massive: 6 (2.4%)		
Location	diffuse: 25 (9.8%)		
	hepatorenal recess: 7 (2.7%)		

splenorenal recess: 4 (1.6%)
around bladder: 29 (11.4%)
other location: 59 (23.1%)

Additional findings (non-exhaustive list)

Intraparenchymal lesions / metastases / masses
Simple and complex cysts
Splenic septic emboli
Uterine abnormalities
Renal infarction
Unintentional duodenal position of gastric tube
Retroperitoneal hematoma
Occlusion of the portal vein
Bowel wall thickening
Para-aortic lymph node metastases
Duplex kidneys
