# Supplementary material for: Development and evaluation of the focused assessment of sonographic pathologies in the intensive care unit (FASP-ICU) protocol
Source: Crit Care. 2021 Nov 24;25:405. doi: 10.1186/s13054-021-03811-2 (PMC8611927; doi:10.1186/s13054-021-03811-2)
Supplement: Supplementary file 4 — Additional file 4. Sonographically assessable abnormalities mentioned in patients’ medical records. [file 13054_2021_3811_MOESM4_ESM.pdf]

Additional file 4

Sonographically assessable pathologies mentioned in patients' medical records

**Were sonographically assessable pathologies mentioned in the patient's medical record?**	yes	72 (n)<br>64.9 (%)	no	39 (n)<br>35.1 (%)
**Did routine WBU confirm the mentioned pathologies or were they at least plausible?**	confirmed or plausible	60 (n)<br>83.3 (%)	unconfirmed or implausible	12 (n)<br>16.7 (%)
**Were additional pathologies detected in patients who had at least one sonographic assessable pathology mentioned in their medical records?**	yes	67 (n)<br>93.1 (%)	no	5 (n)<br>6.9 (%)
