# Supplementary figures and images for: Development and evaluation of the focused assessment of sonographic pathologies in the intensive care unit (FASP-ICU) protocol
Source: Crit Care. 2021 Nov 24;25:405. doi: 10.1186/s13054-021-03811-2 (PMC8611927; doi:10.1186/s13054-021-03811-2)

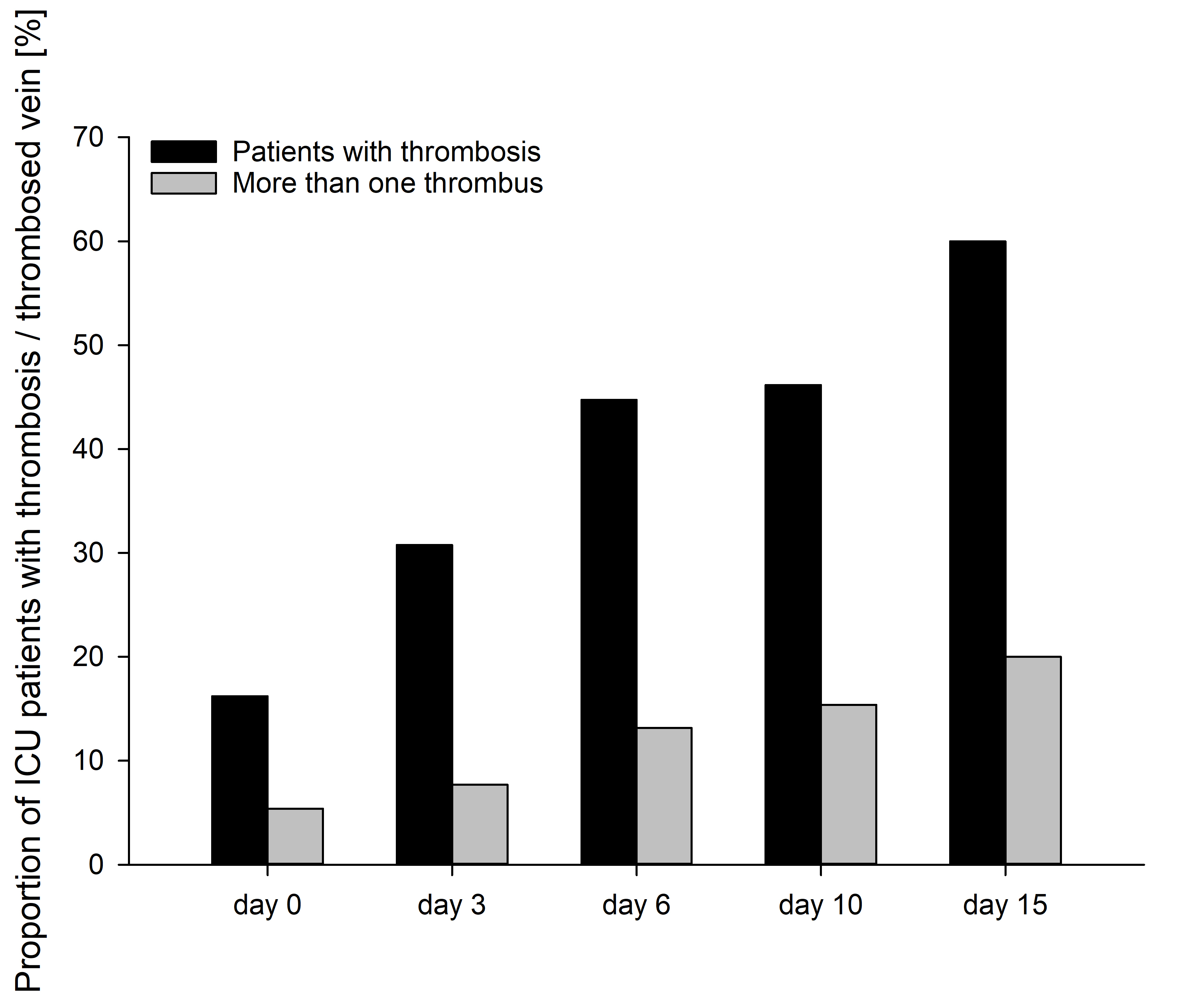

Supplement: Supplementary file 5 — Additional file 5. Incidence of thrombosis detected using the FASP-ICU protocol. [file 13054_2021_3811_MOESM5_ESM.jpg]

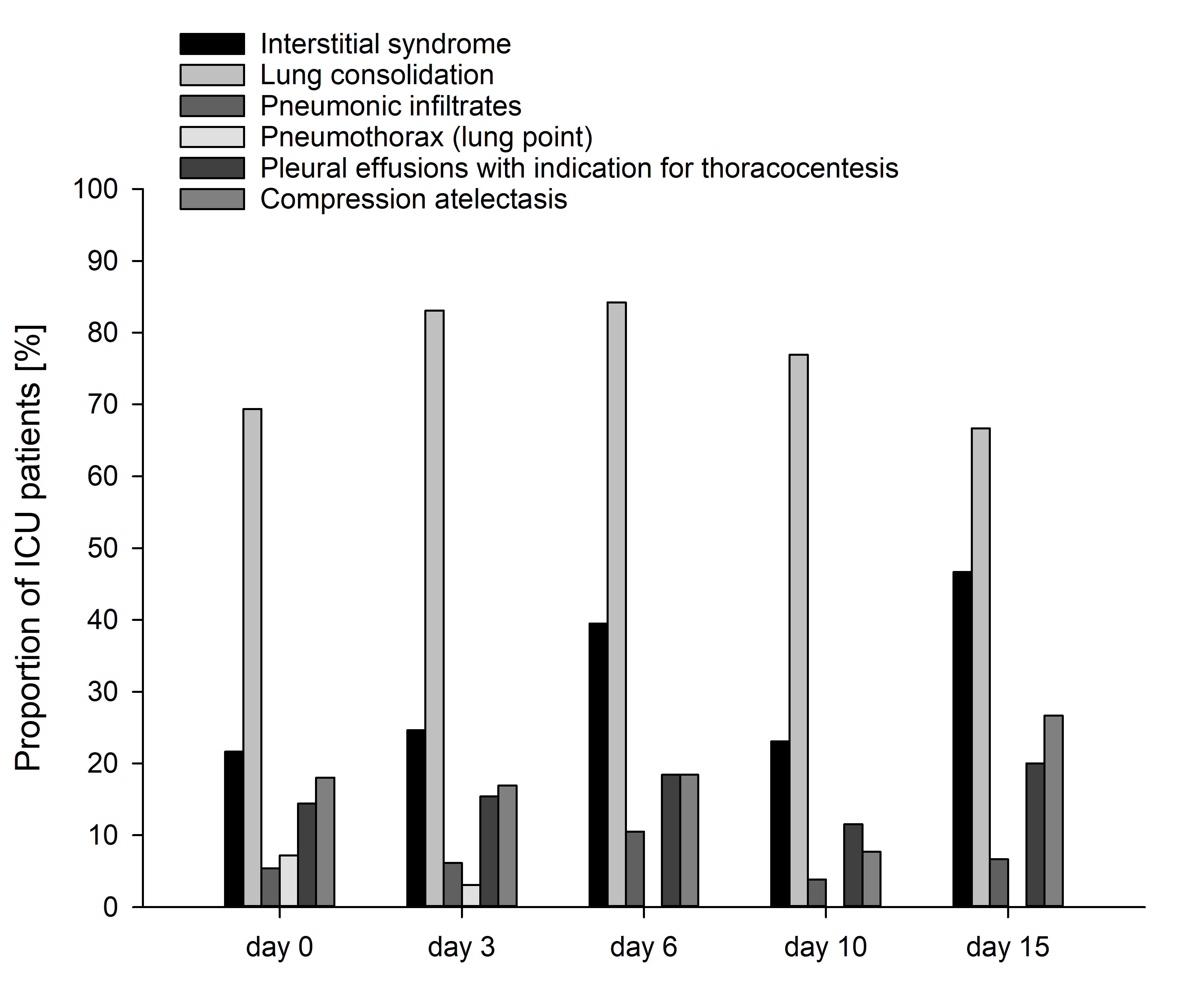

Supplement: Supplementary file 6 — Additional file 6. Proportion of patients with pulmonary sonographic abnormalities detected using the FASP-ICU protocol. [file 13054_2021_3811_MOESM6_ESM.jpg]

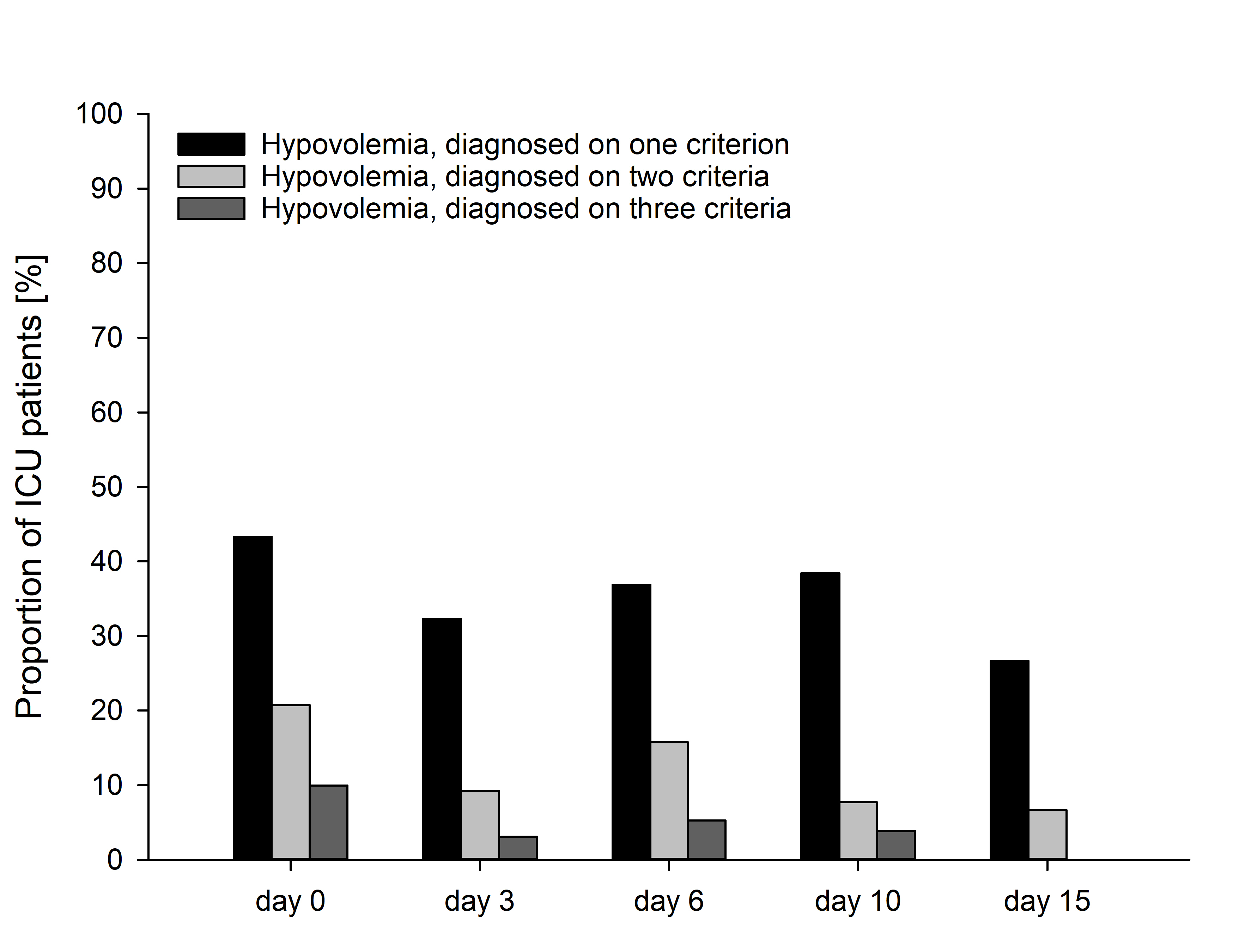

Supplement: Supplementary file 7 — Additional file 7. Proportion of patients with hypovolemia detected using the FASP-ICU protocol. Hypovolemia was assessed according to three criteria: 1 – inferior vena cava (IVC) assessment (respiratory variations in IVC diameter); 2 – right ventricular dimensions and flow measurements; 3 – left ventricular dimensions and flow measurements (intracardiac hypovolemia was diagnosed when ventricular collapse, the papillary muscle kissing sign or more elaborate signs, such as very small end-diastolic areas and velocity time integral (VTI) variations in the left ventricular outflow tract, were present). In the assessment of hypovolemia, single values should be used with care. They are assumed to have low sensitivities and specificities for diagnosing hypovolemia in ICU patients, especially single IVC assessments, which have a number of confounders and limitations. As there are no validated ultrasound criteria for hypovolemia in ICU patients, we recommend using a combination of ultrasound and non-ultrasound assessments until further evidence becomes available. [file 13054_2021_3811_MOESM7_ESM.jpg]

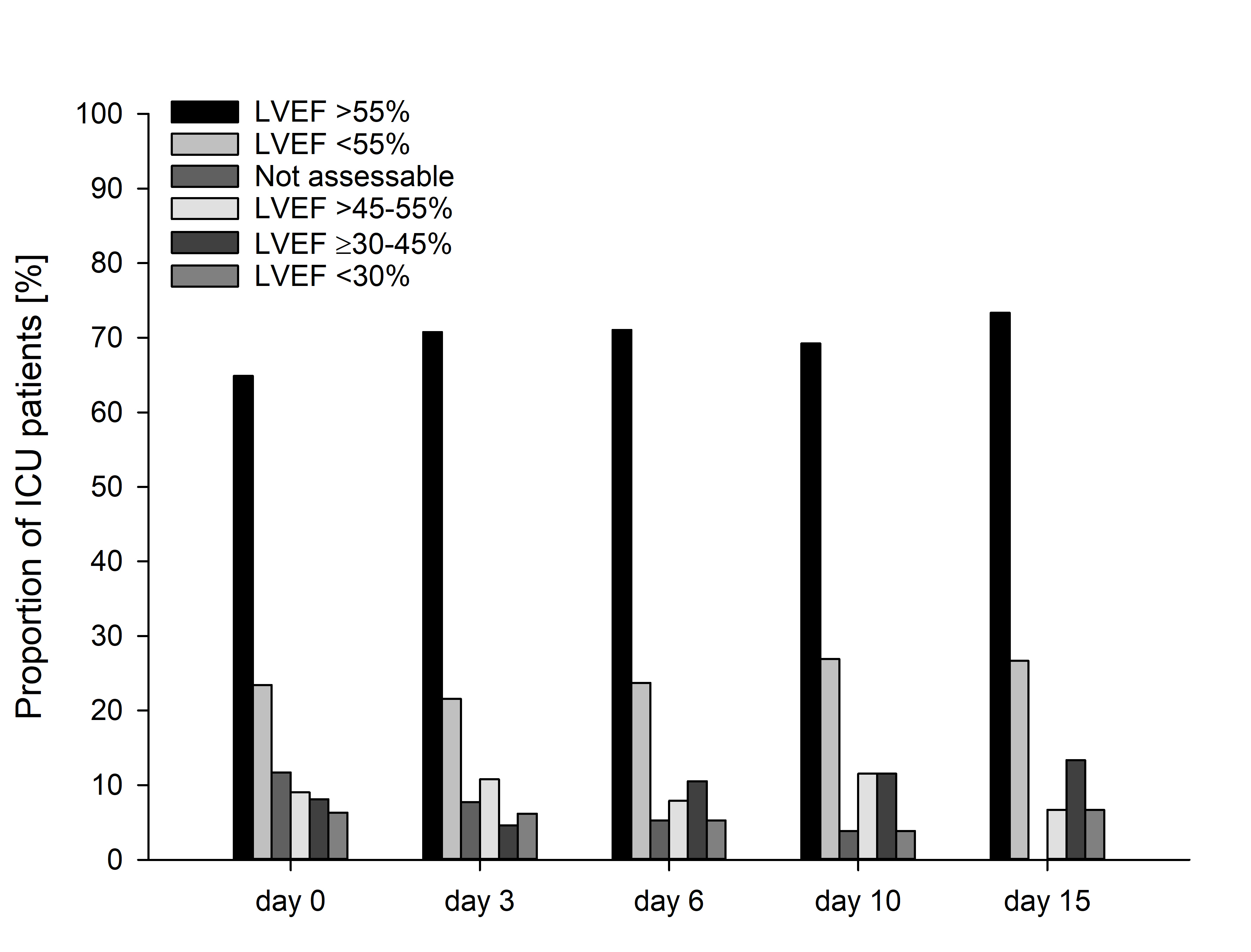

Supplement: Supplementary file 8 — Additional file 8. Assessment of left ventricular ejection fraction (LVEF) by day of scan. [file 13054_2021_3811_MOESM8_ESM.jpg]

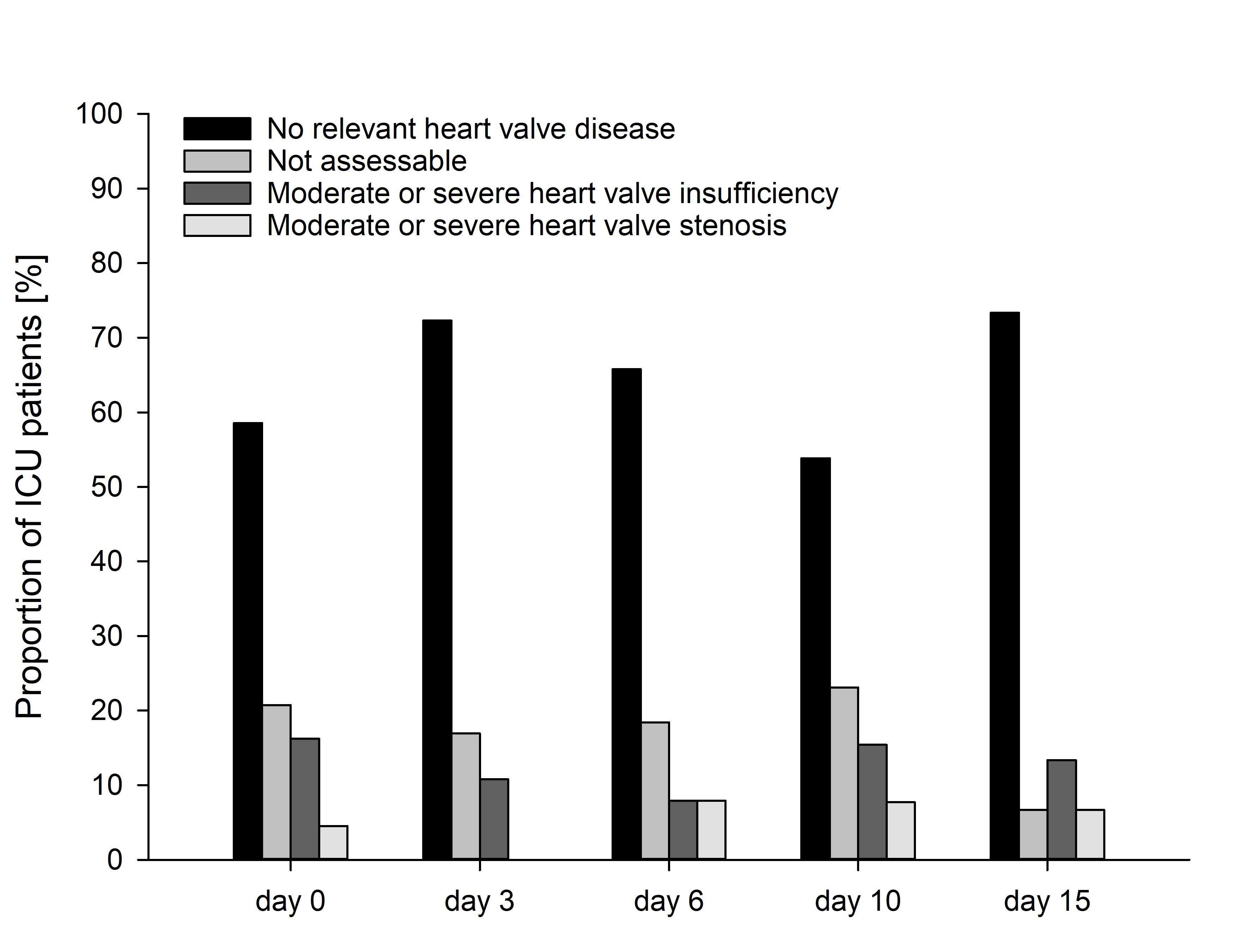

Supplement: Supplementary file 9 — Additional file 9. Presence of heart valve abnormalities detected using the FASP-ICU protocol. [file 13054_2021_3811_MOESM9_ESM.jpg]

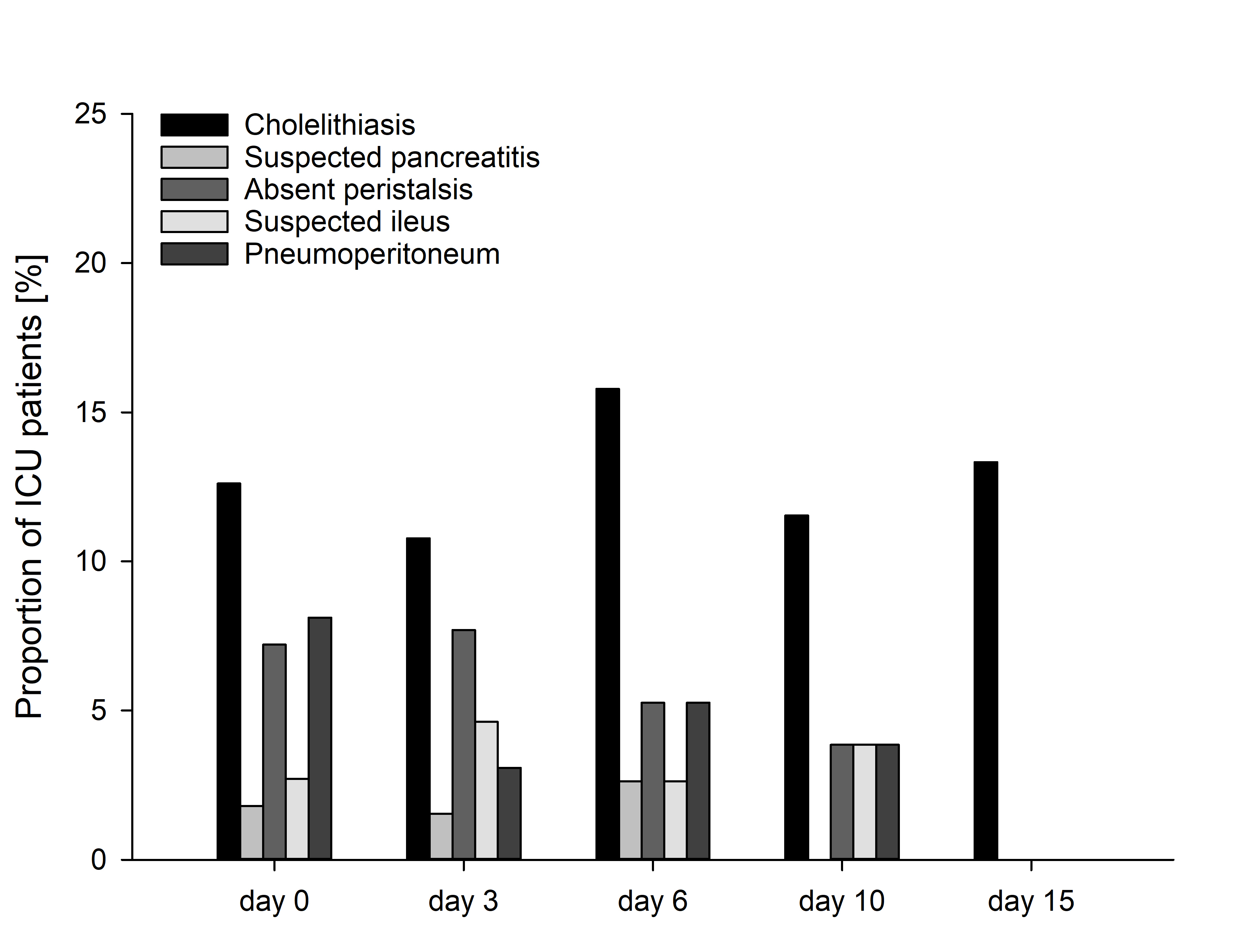

Supplement: Supplementary file 10 — Additional file 10. Presence of common abdominal abnormalities detected using the FASP-ICU protocol. [file 13054_2021_3811_MOESM10_ESM.jpg]

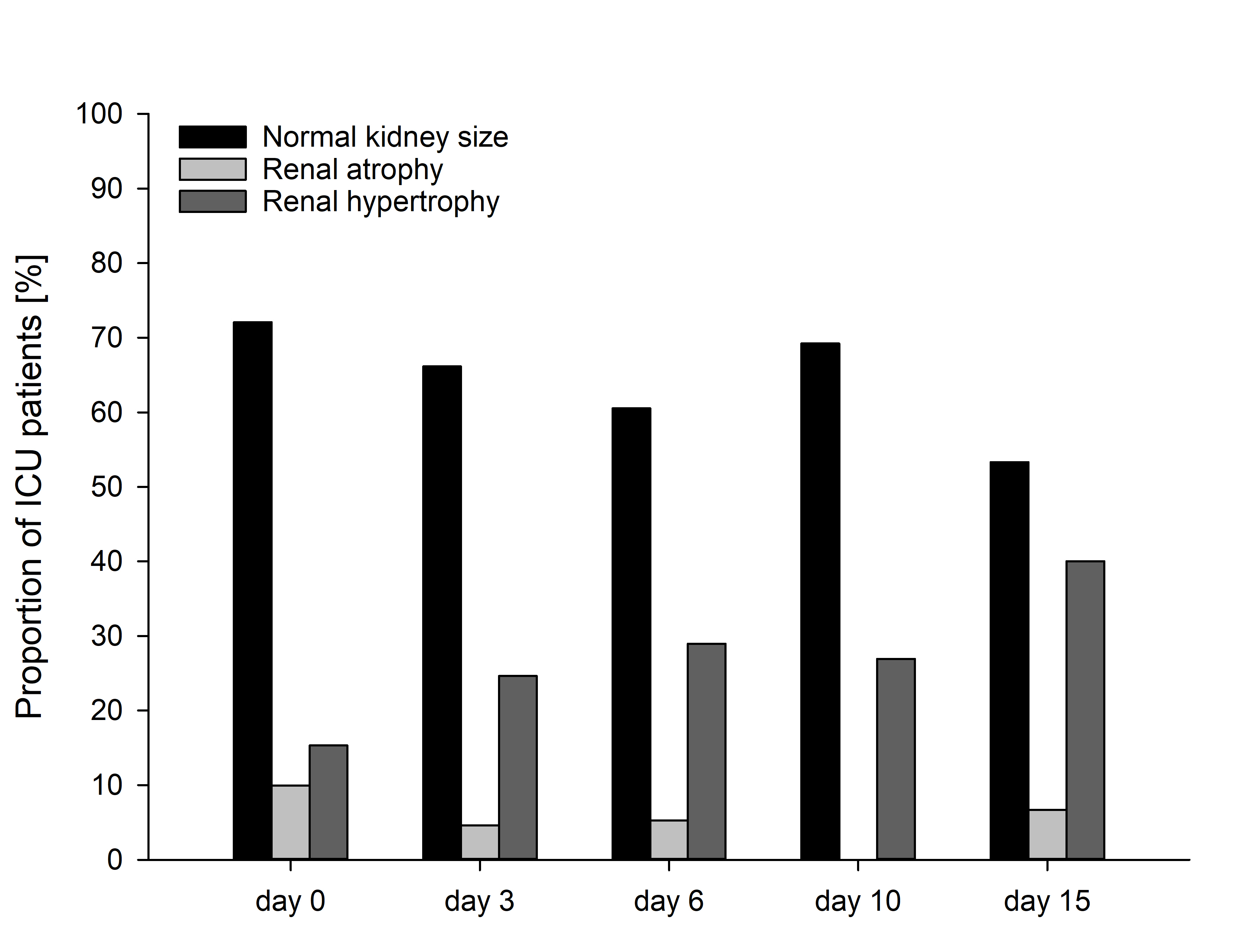

Supplement: Supplementary file 11 — Additional file 11. Presence of renal abnormalities detected using the FASP-ICU protocol. [file 13054_2021_3811_MOESM11_ESM.jpg]

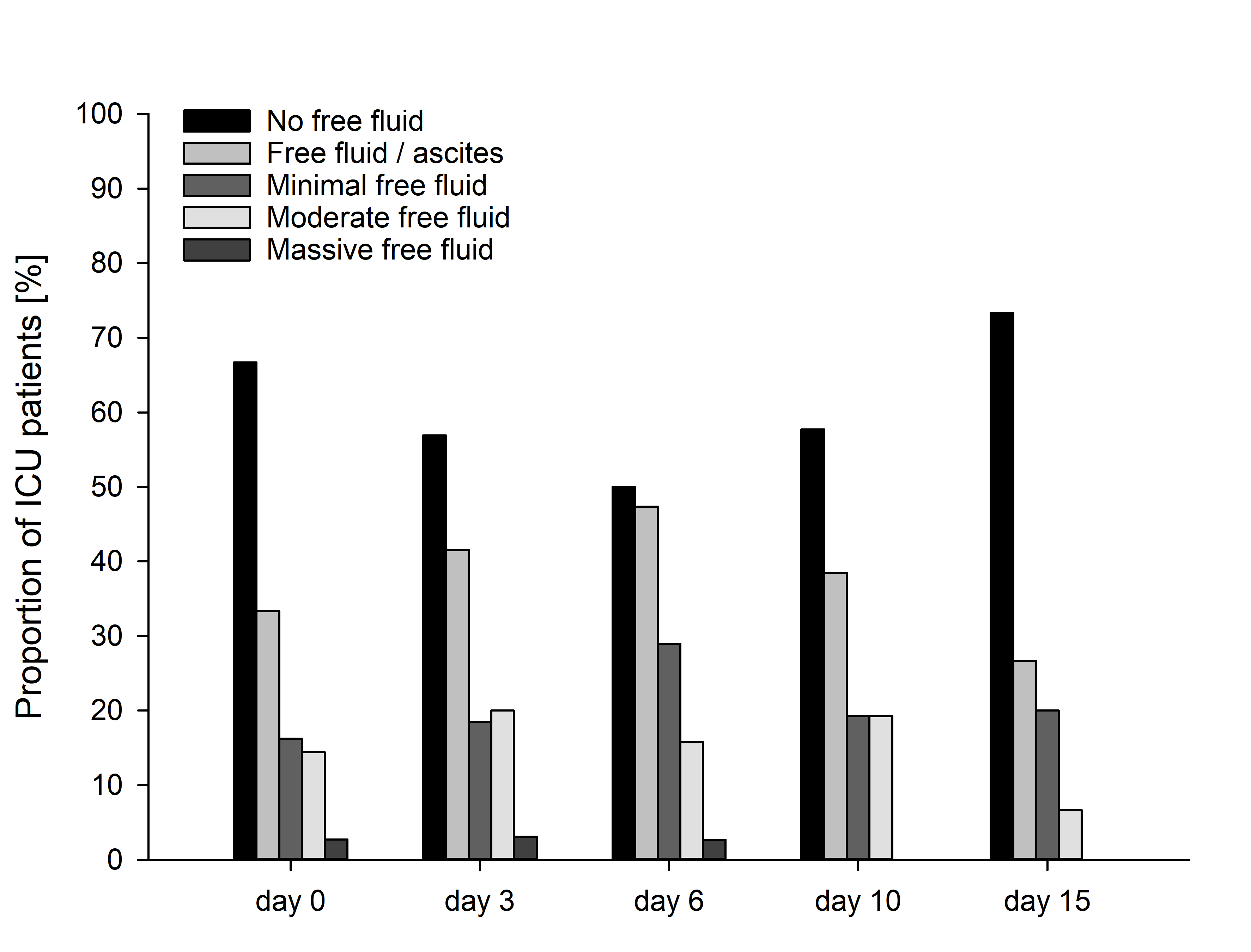

Supplement: Supplementary file 12 — Additional file 12. Presence of intra-abdominal free fluid / ascites detected using the FASP-ICU protocol. [file 13054_2021_3811_MOESM12_ESM.jpg]

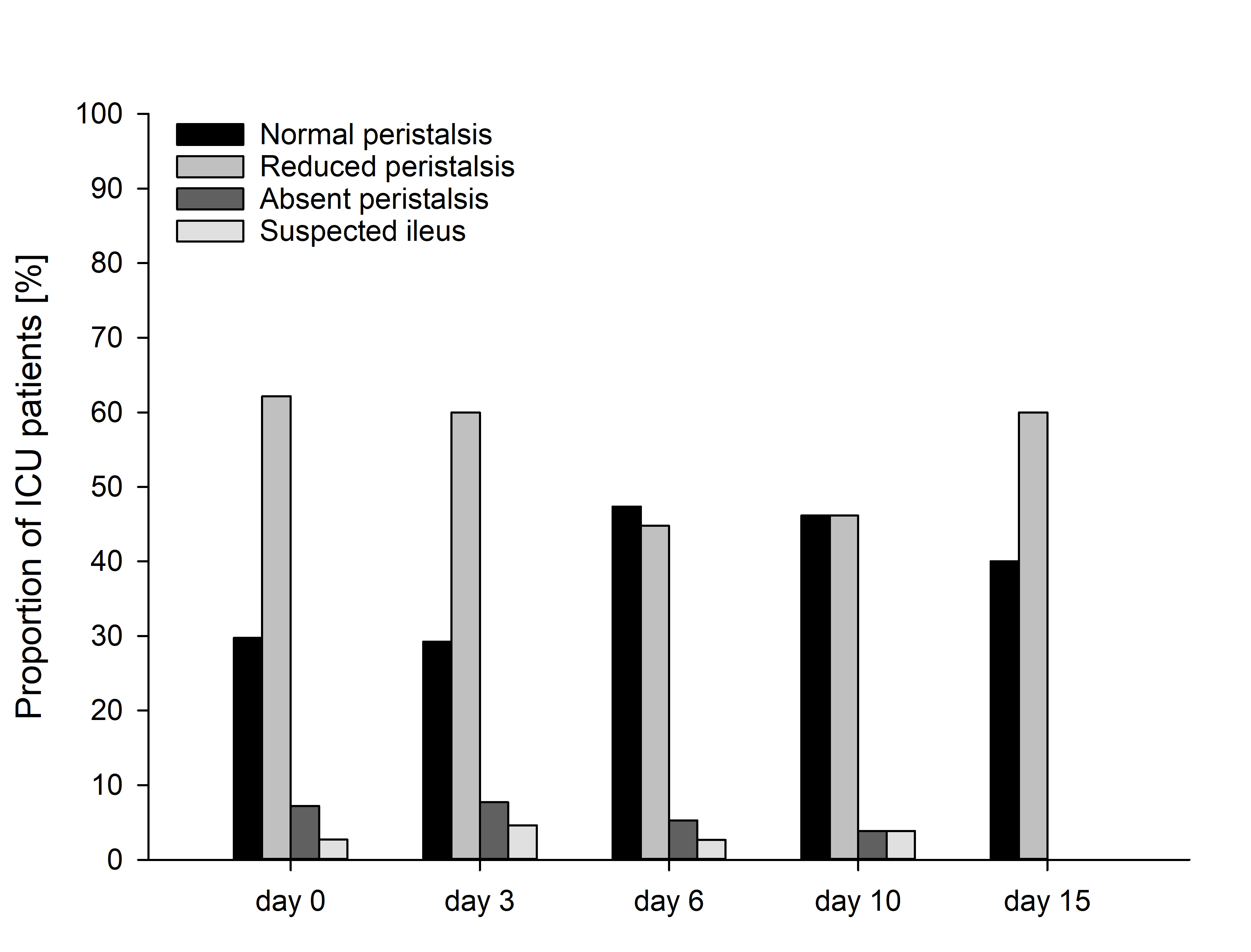

Supplement: Supplementary file 13 — Additional file 13. Presence of peristalsis detected using the FASP-ICU protocol. [file 13054_2021_3811_MOESM13_ESM.jpg]

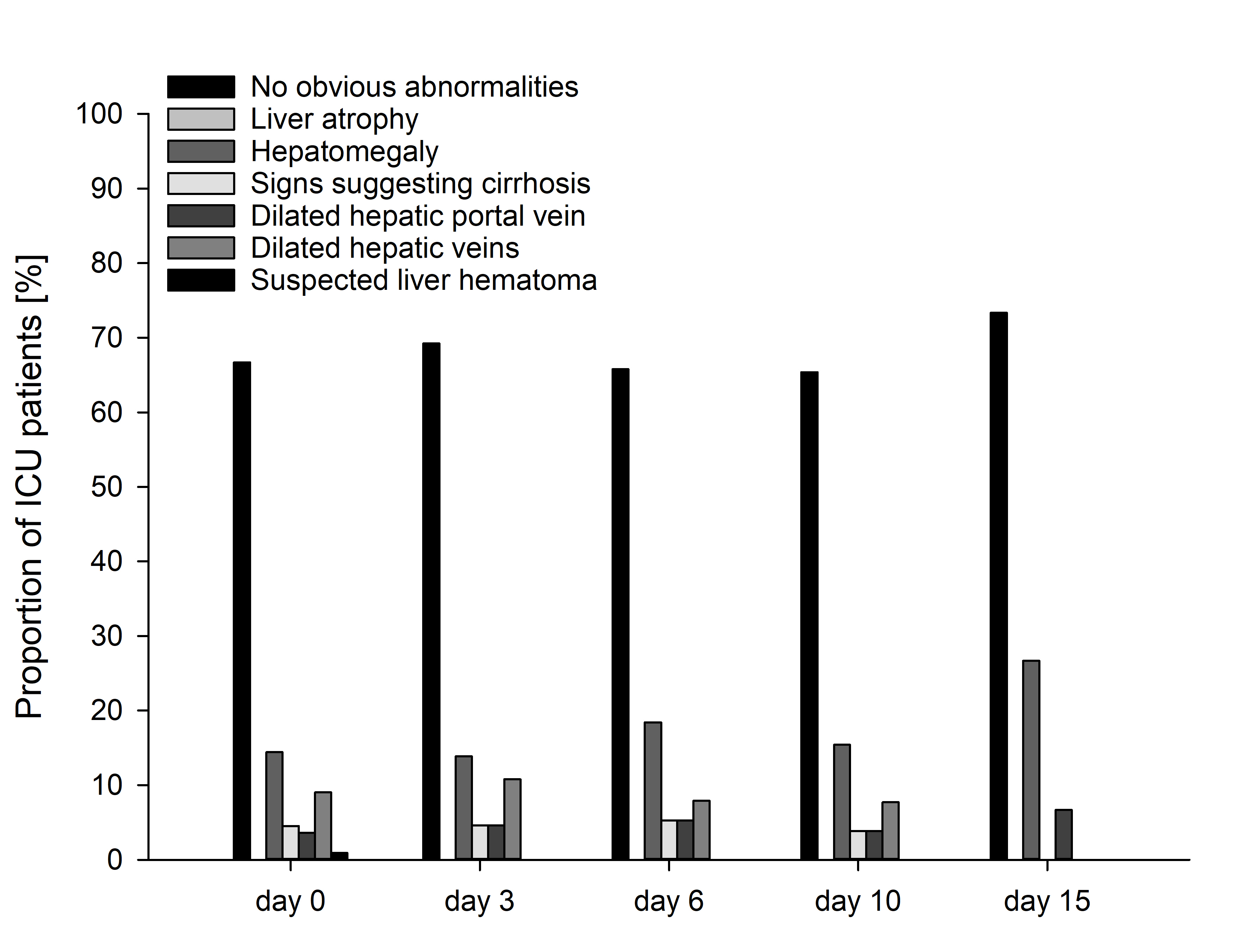

Supplement: Supplementary file 14 — Additional file 14. Proportion of patients showing liver abnormalities detected using the FASP-ICU protocol. [file 13054_2021_3811_MOESM14_ESM.jpg]
