# Supplementary material for: Development and evaluation of the focused assessment of sonographic pathologies in the intensive care unit (FASP-ICU) protocol
Source: Crit Care. 2021 Nov 24;25:405. doi: 10.1186/s13054-021-03811-2 (PMC8611927; doi:10.1186/s13054-021-03811-2)
Supplement: Supplementary file 15 — Additional file 15. Correlations between commonly used ICU parameters and sonographic values. [file 13054_2021_3811_MOESM15_ESM.pdf]

Additional file 15

Correlations between commonly used ICU parameters and sonographic values

SAPS II predicting new sonographic pathologies	$R = 0.401$ $R^2 = 0.160$ adj. $R^2 = 0.152$<br>New pathologies = 1.806 + (0.125·SAPS II)
SAPS II predicting value scale score	$R = 0.207$ $R^2 = 0.043$ adj. $R^2 = 0.032$<br>Value scale score = 4.492 + (0.0424·SAPS II)
SAPS II predicting likelihood of changes to therapy	$R = 0.152$ $R^2 = 0.023$ adj. $R^2 = 0.012$<br>Therapy changes = 0.231 + (0.00593·SAPS II)
Days in intensive care predicting new sonographic pathologies	$R = 0.248$ $R^2 = 0.062$ adj. $R^2 = 0.053$<br>New pathologies = 5.594 + (0.128·days in intensive care)
Days in intensive care pedicting value scale score	$R = 0.148$ $R^2 = 0.022$ adj. $R^2 = 0.012$<br>Value scale score = 5.466 + (0.0896·days in intensive care)
Days in intensive care predicting likelihood of changes to therapy	$R = 0.116$ $R^2 = 0.014$ adj. $R^2 = 0.004$<br>Therapy changes = 0.422 + (0.00723·days in intensive care)
Age predicting new sonographic pathologies	$R = 0.086$ $R^2 = 0.007$ adj. $R^2 = 0.000$<br>New pathologies = 4.527 + (0.0276·age)

R: correlation coefficient; R^2: coefficient of determination; adj. R^2: adjusted R-squared value; SAPS II: Simplified Acute Physiology Score.
