# Supplementary material for: Development and evaluation of the focused assessment of sonographic pathologies in the intensive care unit (FASP-ICU) protocol
Source: Crit Care. 2021 Nov 24;25:405. doi: 10.1186/s13054-021-03811-2 (PMC8611927; doi:10.1186/s13054-021-03811-2)
Supplement: Supplementary file 16 — Additional file 16. Suggestions of protocol additions and alterations that were identified during the review process. They will be evaluated for inclusion in an updated FASP-ICU protocol and should be considered when executing the original version of the FASP-ICU protocol. [file 13054_2021_3811_MOESM16_ESM.pdf]

**Additional file 16**

**Suggestions of protocol additions and alterations identified during the review process. They will be evaluated for inclusion in an updated FASP-ICU protocol and should be considered when executing the original version of the FASP-ICU protocol.**

Ocular ultrasound

Cut-off values for optic nerve sheath diameter (ONSD) as a surrogate for increased intracranial pressure vary in the literature. Depending on personal or institutional needs, a different scientifically evaluated cut-off value can be chosen, which will affect the ratio of sensitivity to specificity.

Protocol addition: Papilledema

In a study with 21 patients by Lochner et al. [38], papilledema out-performed ONSD, as it is easier to detect. The presence of papilledema should be evaluated contemporaneously with ONSD until further evidence from larger trials becomes available.

Focused abdominal ultrasound – liver section

Protocol addition: Presence of portal venous gas

Focused echocardiography – left ventricular ejection fraction

Protocol addition: Hyperdynamic left ventricular ejection fraction

The left ventricular ejection fraction (LVEF) ranges used in this study have been replaced with newer sex-specific ranges and severity cut-off values [39], which should replace the cut-off values used in this study.

Fluid overload and venous congestion assessment

A promising and novel grading system for venous congestion (Venous Excess UltraSound [VExUS] grading system), based on the combination of multiple ultrasound findings, has been developed [40], and should be used when fluid overload and venous congestion are suggested by parameters used in the FASP-ICU protocol.

References

38. Lochner P, Brio F, Zedde ML et al. Feasibility and usefulness of ultrasonography in idiopathic intracranial hypertension or secondary intracranial hypertension. BMC Neurol. 2016 Jun 2; 16: 85. doi: 10.1186/s12883-016-0594-3

39. Roberto M. Lang RM, Badano LP et al. Recommendations for cardiac chamber quantification by echocardiography in adults: An update from the American Society of Echocardiography and the European Association of Cardiovascular Imaging J Am Soc Echocardiogr 2015; 28: 1–39. doi: 10.1016/j.echo.2014.10.003

40. Beaubien-Souligny W, Rola P, Haycock K et al. Quantifying systemic congestion with Point-Of-Care ultrasound: development of the venous excess ultrasound grading system. Ultrasound J 2020; 12: 16. doi: 10.1186/s13089-020-00163-w
